# Role of G-protein coupled receptors in cardiovascular diseases

**DOI:** 10.3389/fcvm.2023.1130312

**Published:** 2023-06-05

**Authors:** Yuanqiang Li, Boyu Li, Wei-Dong Chen, Yan-Dong Wang

**Affiliations:** ^1^ State Key Laboratory of Chemical Resource Engineering, College of Life Science and Technology, Beijing University of Chemical Technology, Beijing, China; ^2^ Key Laboratory of Receptors-Mediated Gene Regulation and Drug Discovery, School of Basic Medical Science, Inner Mongolia Medical University, Hohhot, China; ^3^ Key Laboratory of Receptors-Mediated Gene Regulation, School of Medicine, The People’s Hospital of Hebi, Henan University, Kaifeng, China; ^4^ Department of Gastroenterology and Hematology, The People's Hospital of Hebi, Henan, China

**Keywords:** G-protein-coupled receptors, cardiovascular diseases, vascular tone, ischemia-reperfusion, heart function

## Abstract

Cardiovascular diseases (CVDs) are the leading cause of death globally, with CVDs accounting for nearly 30% of deaths worldwide each year. G-protein-coupled receptors (GPCRs) are the most prominent family of receptors on the cell surface, and play an essential regulating cellular physiology and pathology. Some GPCR antagonists, such as β-blockers, are standard therapy for the treatment of CVDs. In addition, nearly one-third of the drugs used to treat CVDs target GPCRs. All the evidence demonstrates the crucial role of GPCRs in CVDs. Over the past decades, studies on the structure and function of GPCRs have identified many targets for the treatment of CVDs. In this review, we summarize and discuss the role of GPCRs in the function of the cardiovascular system from both vascular and heart perspectives, then analyze the complex ways in which multiple GPCRs exert regulatory functions in vascular and heart diseases. We hope to provide new ideas for the treatment of CVDs and the development of novel drugs.

## Introduction

Cardiovascular diseases (CVDs) are the leading cause of death worldwide. CVDs are divided into two categories, vascular diseases and heart diseases (1). Vascular diseases include hypertension, atherosclerosis, aortic aneurysms, and vascular calciﬁcation (2), while the major components of heart diseases are ischemic heart diseases, rheumatic heart diseases, cardiomyopathy, and myocarditis (3). There are numerous options for treating CVDs, such as lipid-lowering drugs, antihypertensive drugs, antiplatelet and anticoagulant therapies. Despite the effectiveness of these approaches, there is still a long way from curing CVDs (4). Thus, it is crucial to find novel therapeutic targets and develop new drugs to treat CVDs.

G-protein-coupled receptors (GPCRs), which are the most prominent receptor family among all cell surface proteins (5), play essential roles in various human physiological and pathological processes (6). GPCRs contain seven transmembrane α-helices and are coupled with heterotrimeric GTP-binding proteins (G proteins), which are composed of Gα, Gβ, and Gγ subunits. Depending on the difference between Gα subunits, G proteins can be divided into four categories that play different roles. First of all, Gα_s_ can generate the second messenger cyclic-3′,5′-adenosine monophosphate (cAMP) by activating adenylate cyclase, while Gα_i/o_ exerts the opposite effect. Then Gα_q/11_ activates phospholipase C (PLC) to produce the second messenger inositol 1,4,5-trisphosphate (IP3). Finally, Gα_12/13_ can regulate downstream signals through the small GTPase Rho (7).

GPCRs are widely expressed in the cardiovascular system and play crucial roles in regulating cardiovascular function and morphology (8). β-Adrenergic receptors (βARs) and angiotensin II type 1 receptors (AT_1_Rs) are important GPCRs in cardiovascular function. In addition, there are many other GPCRs, such as apelin receptor (APJ), lysophosphatidic acid receptor (LPARs) and endothelin receptors (ET_A_R and ET_B_R), that play important roles in CVDs. Chronic activation by their endogenous ligands increases the workload of the heart, leading to harmful effects such as heart failure (HF) (9). For these reasons, β-blockers and angiotensin-converting enzyme inhibitors are recommended by WHO as essential medicines for patients with CVDs. Furthermore, roughly one-third of all currently used drugs in cardiovascular practice target GPCRs (10). In this review, we summarize the role of GPCRs in CVDs from both vascular diseases and heart diseases, providing new ideas for the treatment of cardiovascular diseases and the development of innovative drugs (Table 1).

**Table 1:** GPCRs in cardiovascular system and cardiovascular disease.

GPCR Family	Receptor	Gα subunits	Ligands	Cell types with expression	Main functions	Clinical agents for CVDs	References
Angiotensin Receptors	AT_1_R	Gα_q/11_ Gα_i/o_ Gα_12/13_	Angiotensin II Angiotensin III	SMCs ECs Cardiomyocyte	Promote vasoconstriction Induce atherosclerosis	Valsartan Olmesartan Losartan Eprosartan Candesartan cilexetil Telmisartan Irbesartan Azilsartan medoxomil	(11–18)
	AT_2_R	Gαi/o			Promote vasoconstriction	N/A	
Adrenoceptors	α1-AR	Gα_q/11_	Norepinephrine Adrenaline	SMCs ECs Cardiomyocyte	Promote vascular and cardiac contraction	Labetalol Ergoloid Mephentermine Methoxamine Midodrine Phentolamine Tolazoline	(19–44)
	α2-AR	Gα_i/o_			Promote atherosclerosis Inhibit cardiac contraction	Labetalol Ergoloid Mephentermine Metaraminol Phenoxybenzamine Phentolamine Tolazoline	
	β1-AR	Gα_s_ Gα_i/o_		SMCs Cardiomyocyte	Mediate vasodilation Promote cardiac contraction	Carvedilol Betaxolol Metoprolol Atenolol Timolol Bisoprolol Mephentermine	
	β2-AR	Gα_s_ Gα_i/o_			Promote cardiac contraction	Carvedilol Carteolol Mephentermine Sotalol	
	β3-AR	Gα_i/o_		Cardiomyocyte	Inhibit cardiac contraction	Norepinephrine Propranolol Labetalol Mephentermine	
Apelin Receptor	APJ	Gα_q/11_ Gα_i/o_	Apelin Apela	SMCs ECs Cardiomyocyte	Promote vascular and cardiac contraction Induce vasodilatation Response shear stress Promote atherosclerosis Maintain normal heart development	Apelin (In Trial)	(19, 45–51)
Lysophosphatidic Acid (LPA) Receptors	LPAR1	Gα_q/11_ Gα_i/o_	Lysophosphatidic Acid	SMCs ECs Cardiomyocyte	Induce vasorelaxation Cause vasoconstriction Accelerate atherosclerosis	N/A	(52–58)
	LPAR2	Gα_q/11_ Gα_i/o_ Gα_12/13_		SMCs ECs	Accelerate atherosclerosis	N/A	
	LPAR3	Gα_q/11_ Gα_i/o_		SMCs ECs Cardiomyocyte	Accelerate atherosclerosis	N/A	
	LPAR5	Gα_q/11_ Gα_12/13_			Induce early atherosclerosis	N/A	
	LPAR6	Gα_12/13_		ECs	Accelerate atherosclerosis	N/A	
Endothelin Receptors	ET_A_R	Gα_q/11_ Gα_i/o_	Endothelin-1 Endothelin-2 Endothelin-3	SMCs ECs Cardiomyocyte	Promote vasoconstriction Accelerate atherosclerosis	Bosentan Ambrisentan Macitentan	(59–63)
	ET_B_R				Promote vasodilation		
Purinergic Receptors	P_2_Y_2_	Gα_q/11_	Low-density lipoprotein	SMCs ECs Cardiomyocyte	Promote vascular inflammation Accelerate atherosclerosis	Promethazine	(64–66)
	P_2_Y_6_	Gα_q/11_ Gα_12/13_	ADP		Accelerate atherosclerosis		
Proteinase-activated Receptors	PAR1	Gα_q/11_ Gα_12/13_	Thrombin	SMCs ECs Cardiomyocyte	Lead to endothelial dysfunction	Vorapaxar	(67, 68)
	GPR75	Gα_q/11_	20-hydroxyeicosatetraenoic	ECs	Cause vasoconstriction	N/A	(69)
	GPR41	Gα_i/o_ Gα_12/13_	Short-chain fatty acids	SMCs ECs Cardiomyocyte	Induce vasodilation Decrease blood pressure	N/A	(70)
	GPR120	Gα_q/11_ Gα_s_	Omega-3 fatty acid	SMCs ECs Cardiomyocyte	Inhibit oxidative stress and inflammation	Fish oil	(71, 72)
	GPR68	Gα_s_		ECs	Shear stress sensor	N/A	(73, 74)
Histamine Receptors	H_1_R	Gα_q/11_	Histamine	SMCs ECs Cardiomyocyte		Tolazoline Quinidine	(75–77)
Chemokine Receptors	CXCR4	Gα_q/11_ Gα_i/o_	MIF	ECs Cardiomyocyte	Promote vascular inflammation Accelerate atherosclerosis	Baclofen	(78, 79)
	CCR2	Gα_q/11_	CCL2	SMCs ECs		N/A	(80)
	CX_3_CR1		CX_3_CL1	SMCs ECs Cardiomyocyte		N/A	(81)
Thromboxane A2 receptor	TP	Gα_q/11_	Thromboxane A2	SMCs ECs Cardiomyocyte		N/A	(82)
Glucagon Receptor	GLP-1R	Gα_s_	Glucagon-like peptide	SMCs ECs Cardiomyocyte		Liraglutide Dulaglutide Albiglutide Semaglutide	(83–86)
Sphingosine 1-phosphate Receptors	S1PR1	Gα_i/o_	Sphingosine 1-phosphate	SMCs ECs Cardiomyocyte	Promote the normal development of the heart	N/A	(87–89)
Prokineticin Receptors	PKR1	Gα_q/11_ Gα_i/o_	Prokineticin-1 Prokineticin-2	ECs Cardiomyocyte		N/A	(90, 91)
Chemokine Receptors	CXCR7		CXCL12	SMCs Cardiomyocyte		N/A	(92, 93)
Corticotropin-releasing factor Receptors	CRH-R2	Gα_s_	CRH	SMCs ECs Cardiomyocyte	Promote heart contraction	N/A	(94)
Opioid Receptors	δ receptor	Gα_i/o_	β-endorphin	SMCs ECs Cardiomyocyte	Protect the heart during ischemia-reperfusion	N/A	(95–99)
	κ receptor	Gα_i/o_				N/A	
	μ receptor	Gα_i/o_				N/A	
Adenosine Receptors	A_1_	Gα_i/o_	Adenosine	SMCs ECs Cardiomyocyte	Protect heart	Adenosine Theophylline Theobromine Binodenoson	(100–107)
	A_2A_	Gα_s_					
	A_2B_	Gα_s_					
	A_3_	Gα_i/o_					
Calcium-sensing receptors	CaSR	Gα_q/11_ Gα_i/o_ Gα_12/13_ Gα_s_	Ca^2+^	SMCs ECs Cardiomyocyte	Exacerbate ischemia-reperfusion injury in the heart	N/A	(108, 109)

## GPCRs in vascular function and disease

### GPCRs and vascular function

Vascular homeostasis is essential for maintaining the health of the body. Smooth muscle cells (SMCs) are a major structural component of the vessel wall, regulating vascular tone to maintain intravascular pressure (110). Meanwhile, endothelial cells (ECs) are critical regulators of vascular inflammation, thrombophlebitis, permeability, and vascular remodeling (111). Under normal conditions, SMCs and ECs exert a protective role, maintaining vascular stability. However, during the development of vascular disease, the dysfunction of ECs and the dedifferentiation of SMCs promote pathological changes in the vasculature, thereby accelerating the process of vascular diseases. Single-cell GPCR expression analysis demonstrates that the expression of GPCRs in ECs and SMCs is highly heterogeneous. Vascular diseases such as atherosclerosis lead to characteristic changes in the expression of GPCRs (112). Thus, in the vascular system, GPCRs are critical regulators.

GPCRs regulate blood pressure by modulating the dynamic balance of vasoconstriction and relaxation (113, 114). Gα_q/11_-coupled GPCRs and Gα_12/13_-coupled GPCRs cause vasoconstriction via Ca^2+^ and RhoA, respectively. Conversely, Gα_s_-coupled GPCRs can generate cAMP, and then promote blood vessel relaxation (115, 116). One typical example is that angiotensin II (AngII) binds to and activates angiotensin receptors (AT_1_R and AT_2_R), causing the smooth muscle to contract (11). And α1-adrenergic receptor (α1-AR), mainly expressed in SMCs, has a similar function (117). The APJ is highly expressed in cardiovascular tissues, and the apelin/APJ system is vital for regulating vascular tone (45, 46). Apelin/APJ system can inhibit the BKCa signaling pathway (118), increase the phosphorylation of MLC (119), or cooperate with α1-AR to promote vasoconstriction (19). However, the apelin/APJ system can induce vasodilatation by stimulating the release of nitric oxide (NO) (47, 120). This different regulation depends on the type of blood vessels and pathological condition (45). Besides, many other GPCRs are implicated in the regulation of vasoconstriction and relaxation. LPA stimulates LPA receptor 1 (LPAR1), then activates PLC and releases NO to induce vasorelaxation. In addition, activation of LPAR1 can also produce thromboxane A2 (TxA2), which can bind to prostaglandin receptors, leading to vasoconstriction (52–54). The endothelin system includes two GPCRs: endothelin receptor A (ET_A_R) and B (ET_B_R). Endothelin 1 (ET-1) can promote vasoconstriction by activating ET_A_R or promote vasodilation by activating ET_B_R (59). Recent studies have shown that the binding of the orphan receptor GPR75 to 20-hydroxyeicosatetraenoic acid (20-HETE) activates the Gα_q/11_ protein, which causes vasoconstriction (69). And short-chain fatty acids (SCFAs) can activate GPR41 to induce vasodilation (70). In conclusion, GPCRs are critical regulators of vascular tone.

Normal vascular endothelial function is highly crucial for vascular homeostasis. Endothelial dysfunction leads to the destruction of cell connections, vascular leakage, tissue edema, and organ failure (111, 121). Vascular endothelial dysfunction is caused by inflammation, and GPCRs play an essential role in this process (122). Multiple GPCRs agonists, including thrombin, histamine, and prostaglandin E2, stimulate robust p38 autophosphorylation to promote endothelial inflammatory responses (123). Purinergic GPCRs (P_2_Ys) are widely expressed in the cardiovascular system. P_2_Y_1_, P_2_Y_2_, P_2_Y_4_, and P_2_Y_6_ can promote vascular inflammation and reduce endothelial barrier function through the Gα_q_-PLC pathway (124). Protease-activated receptors (PARs) are speciﬁc GPCRs that can be cleaved by serine proteases thrombin or trypsin and then regulate downstream signaling pathways. The sustained activation of PAR1 promotes the disruption of endothelial junction proteins, increases endothelial permeability and plasma extravasation, and leads to endothelial dysfunction (67). In contrast, activation of GPR120, a recently identified omega-3 fatty acid receptor, inhibits oxidative stress and inflammation by suppressing the production of reactive oxygen species (ROS) and the expression of pro-inflammatory cytokines. It also can protect vascular endothelial function by preventing monocyte attachment to endothelial cells (71).

The flow of blood causes shear stress, which is a mechanical stimulus. Mechanical stimuli can be sensed by cells and converted into biochemical signals to inspire diverse cellular functions (125, 126). Some GPCRs are initial sensors of mechanical stimulation, they can be activated by shear stress to regulate downstream signals. For instance, GPR68, a mechanosensor expressed in ECs, is significantly responsive to shear stress and is required for ECs' shear stress sensitivity. After the absence of GPR68 in mice, the vasodilation response brought about by the increase in blood flow was disrupted, suggesting that GPR68 is involved in flow-mediated vasodilation and remodeling (73, 74). Similar to GPR68, the H_1_ histamine receptor (H_1_R) is mechanosensitive Gα_q/11_ coupled GPCR highly expressed in ECs. Shear stress activates H_1_R in an agonist-independent manner, leading to vasodilation (75). APJ is another GPCR that can be activated by mechanical stimulation. Flow-induced signaling through APJ is crucial for cell morphology, endothelial elasticity, and cellular adhesion. Deleting APJ not only impairs the elasticity and cell adhesion of ECs but also alters the remodeling of actin filaments and the distribution of vinculin particles (48).

In summary, GPCRs play an important role in regulating vascular tension, maintaining vascular endothelial barrier function, and sensing blood flow shear stress.

### GPCRs and atherosclerosis

Atherosclerosis is a progressive disease. The accumulation of lipids, fibers, or cell debris on the arterial intima interferes normal function of blood vessels and impedes blood flow. In severe cases, it can lead to myocardial infarction or stroke (127). Atherosclerosis can be seen as a response to injury and is a chronic inflammatory disease of blood vessels. Endothelial dysfunction caused by vascular inflammation initiates the process of atherosclerosis. In the presence of inflammatory factors, SMCs migrate to the vascular intima and then proliferate, resulting in atherosclerotic plaques (128, 129). Endothelial dysfunction and the proliferation and migration of SMCs are the fundamental factors of atherosclerosis. Many GPCRs play an essential role in vascular endothelial dysfunction, therefore significantly influencing on the atherosclerotic process.

The role of P_2_Ys in atherosclerosis can be anticipated due to their role in endothelial dysfunction (130). A typical example is P_2_Y_2_. The ATP released by oxidized low-density lipoprotein (LDL) activates P_2_Y_2_, therefore promoting vascular inflammation, ensuring penetration and adhesion of monocytes, and accelerating the process of atherosclerosis (64). In addition, P_2_Y_6_ is upregulated in atherosclerotic lesions, suggesting that it may also promote atherosclerosis. Due to reduced vascular inflammation, P_2_Y_6_-deficient mice have a slowed atherosclerotic process (65). LPA accumulates in atherosclerosis, and the expression of LPAR1-LPAR6 in human arterial plaques and normal arteries is significantly different, suggesting that LPARs may play a role in atherosclerosis (131, 132). Activation of LPAR1 and LPAR3 can promote the expression of hypoxia-inducible factor 1 subunit alpha (HIF-1α), then upregulate C-X-C motif chemokine ligand 1 (CXCL1) in cells, thereby accelerating atherosclerosis (133). LPAR6 can induce actin stress crack formation through the RhoA/ROCK pathway to increase endothelial permeability and advance the occurrence of atherosclerosis (55). Furthermore, LPAR5 activates the TGFBR1, which stimulates the glycosaminoglycan (GAG) chain elongation, resulting in the early pathogenesis of atherosclerosis (56). The interaction between chemokines and their GPCR-type receptors (CRKs) is an element that promotes atherosclerosis. CXC-motif chemokine receptor-4 (CXCR4) (78), CC chemokine receptor 2 (CCR2) (80), and C-X3-C Motif Chemokine Receptor 1 (CX3CR1) (81) accelerate atherosclerosis by promoting vascular inflammation.

The proliferation and migration of SMCs accelerate the occurrence of atherosclerosis, and GPCRs also regulate this process. LPARs play a vital role in the dedifferentiation, proliferation, and migration of SMCs (52). LPA promotes the dedifferentiation of SMCs through LPAR3 (57) and promotes SMC proliferation and migration through LPAR1 and LAPR2 (58). And in-depth studies suggest that LPA may accelerate the atherosclerosis process by activating Gα_q/11_-coupled GPCRs to promote the proliferation and migration of SMCs (134, 135). Yes-associated protein (YAP) signaling pathway is a crucial regulator of the proliferation and migration of SMCs (136). Depending on the difference of the coupled G protein, GPCRs have different effects on the regulation of YAP. Briefly, Gα_i/o_, Gα_q/11_, and Gα_12/13_ can activate YAP, while Gα_s_ exerts an inhibitory effect (137). For instance, the thromboxane A2 receptor (TP) (82), AT_1_R (138), and ET_A_R (60) can activate YAP, then promote the proliferation and migration of SMCs. Activation of APJ by apelin promotes the proliferation of SMCs, while the knockout of APJ reduces the production of ROS and the formation of atherosclerosis (49). By promoting the activity of growth factor receptors EGFR and HGFR, the α2-adrenergic receptor (α2-AR) promotes the proliferation of SMCs cells (20). Furthermore, the Glucagon-like peptide 1 receptor (GLP-1R) is located in the nucleus of rat SMCs, and artificially keeping it in the cytoplasm can promote the proliferation of SMCs (83). Together, these studies show that GPCRs are of profound significance to the occurrence and development of atherosclerosis, and the development of drugs targeting GPCRs to treat atherosclerosis is very necessary.

## GPCRs in heart function and disease

### GPCRs and heart function

The heart principal function is to pump blood to the circulation of the various organs and systems of the human body to achieve the purpose of oxygen supply and nutrient exchange. The normal development of the heart is of great importance. Abnormal heart development leads to heart malformations and congenital heart disease, affecting human life and health (139, 140). After years of research, a large number of pathways and processes that play a regulatory role in the development of the heart have come to light. Among them, GPCRs are regulators that cannot be neglected (90). Sphingosine 1-phosphate (S1P) is a lipid with biological activity, and the activation of its receptors S1P receptors (S1PRs) is essential in the normal development of the heart (87). Mice with global loss of S1PR1 will die 12.5–14.5 days post-coitus due to cardiovascular defects (88). In addition, knocking out S1PR1 in mouse cardiomyocytes will affect standard ventricular compaction, septation, and embryo survival, indicating that S1PR1 in cardiomyocytes is required for the normal development of the heart (89). The apelin-APJ system is an essential regulator of the cardiovascular system. Loss of APJ leads to abnormal development of myocardial progenitor and defects in heart development (50, 51). Apela is an endogenous ligand of APJ newly discovered in recent years (141), and the absence of apela gene also leads to early deformation of heart development (142, 143). Many other GPCRs are also important in heart development. Prokineticin receptor-1 (PKR1) (90), C-X-C motif chemokine receptor 7 (CXCR7) (92, 93), 5-hydroxytryptamine receptor 2B (5-HT_2B_) (144), and atypical chemokine receptor (ACKR) (145) are critical to the development of the heart, and the absence of either of them leads to incomplete heart development and thus death in mice.

The heart's contraction is a complex process involving action potentials, contractile proteins, and excitation-contraction coupling, which has been thoroughly reviewed by predecessors (146–150). Heart contractility is extremely important to the pumping function of the heart, and the decline of contractility can lead to HF, which results in sudden death (147). GPCRs expressed in the human myocardium have both positive and negative regulatory effects on heart contractility. AR family mainly includes five receptors, α1, α2, β1, β2, and β3 (117). These five receptors are all expressed in the heart, and the regulatory role of βARs in the heart is crucial. β1-AR accounts for about 80% of the βARs in the heart, followed by β2-AR, accounting for 15%–18%, and the remaining β3-AR (10). Activation of β1-AR or β2-AR will activate Gα_s_ protein and promote the production of cAMP. Then cAMP acts on protein kinase A (PKA), thereby causing heart contraction (21, 151). Conversely, activation of β3-AR promotes cardiac relaxation through the release of NO (22). Besides, α1-AR (23) and α2-AR (24) perform functions that promote or inhibit cardiac contraction, respectively. In addition, using corticotropin-releasing hormone (CRH) to activate CRH receptor 2 (CRH-R2) in mice can promote heart contraction through a variety of signaling pathways, including adenylate cyclase, PKC, and PKA (94). And the binding of myosuppressin (MS) to its receptor can decrease heart contractility to a great extent (152). In summary, the strategic role of GPCRs in heart development and contraction is evident.

### GPCRs and cardiac ischemia-reperfusion injury

Ischemic heart disease occupies an essential position in all types of heart disease, and its fatality rate has reached almost half of all CVDs, and it is the leading cause of death around the world (3). The ischemia-reperfusion (IR) process is a pathological phenomenon, which refers to first restricting the blood supply to the organ, then restoring the perfusion and corresponding oxygen supply (153). Heart IR will cause many deaths of cardiomyocytes and induce severe autoimmune responses, which may lead to long-term cardiac dysfunction (153, 154). Therefore, effective interventions to limit IR injury (IRI) are critical to protecting the heart. GPCRs have been proven to play a significant role in inhibiting IRI and protecting the heart. The opioid receptor (OR) family is a cardioprotective system, and opioid preconditioning has shown a strong protective effect on IRI (155). δ opioid receptors (DOR) and κ opioid receptors (KOR) are expressed in human cardiomyocytes, while the expression of μ opioid receptors (MOR) is dependent on species (95). During IR, ORs are vital determinants of ischemia and hypoxia tolerance; opioid levels are upregulated in heart ischemic, which leads to the activation of ORs and induces cardioprotective responses (96). ORs preconditioning effects activate a series of downstream signal pathways through the Gα_i/o_-PKC pathway to protect mitochondrial function, inhibit cell death signals, and achieve the purpose of protecting the heart (96–99). Adenosine receptors are another GPCR family that can protect the heart in IR. Studies have shown that the four subtypes of adenosine receptors, A_1_, A_2A_, A_2B,_ and A_3_, have beneficial effects in protecting the heart (156). Activating A_1_ and A_3_ adenosine receptors before ischemia can initiate the ischemic preconditioning response, improve the ischemia tolerance of the heart, and avoid heart damage (100, 101). At the same time, the A_2_ adenosine receptors protect the heart during reperfusion, and the synergistic effect of A_2A_ and A_2B_ may play a non-negligible role in avoiding reperfusion injury (102, 103). S1P is released in the ischemic damaged heart and then binds to S1PRs to protect the heart from IRI by the Gα_12/13_-RhoA-protein kinase D (PKD) pathway (157, 158). However, some GPCRs can exacerbate the IRI of the heart, and the most prominent example is the calcium-sensing receptor (CaSR). CasR is widely expressed throughout the body, and its primary function is to maintain a constant concentration of extracellular ionized Ca^2+^ (108). Research has shown that the activation of CaSR by IR induces mitochondrial apoptosis, which promotes cardiomyocyte apoptosis, causing heart damage (109). In short, GPCRs have a critical role in the positive and negative regulation of IRI. Targeting GPCRs to prevent heart damage, alleviate heart disease, and avoid HF is a promising treatment.

## Conclusion

**Figure 1:**
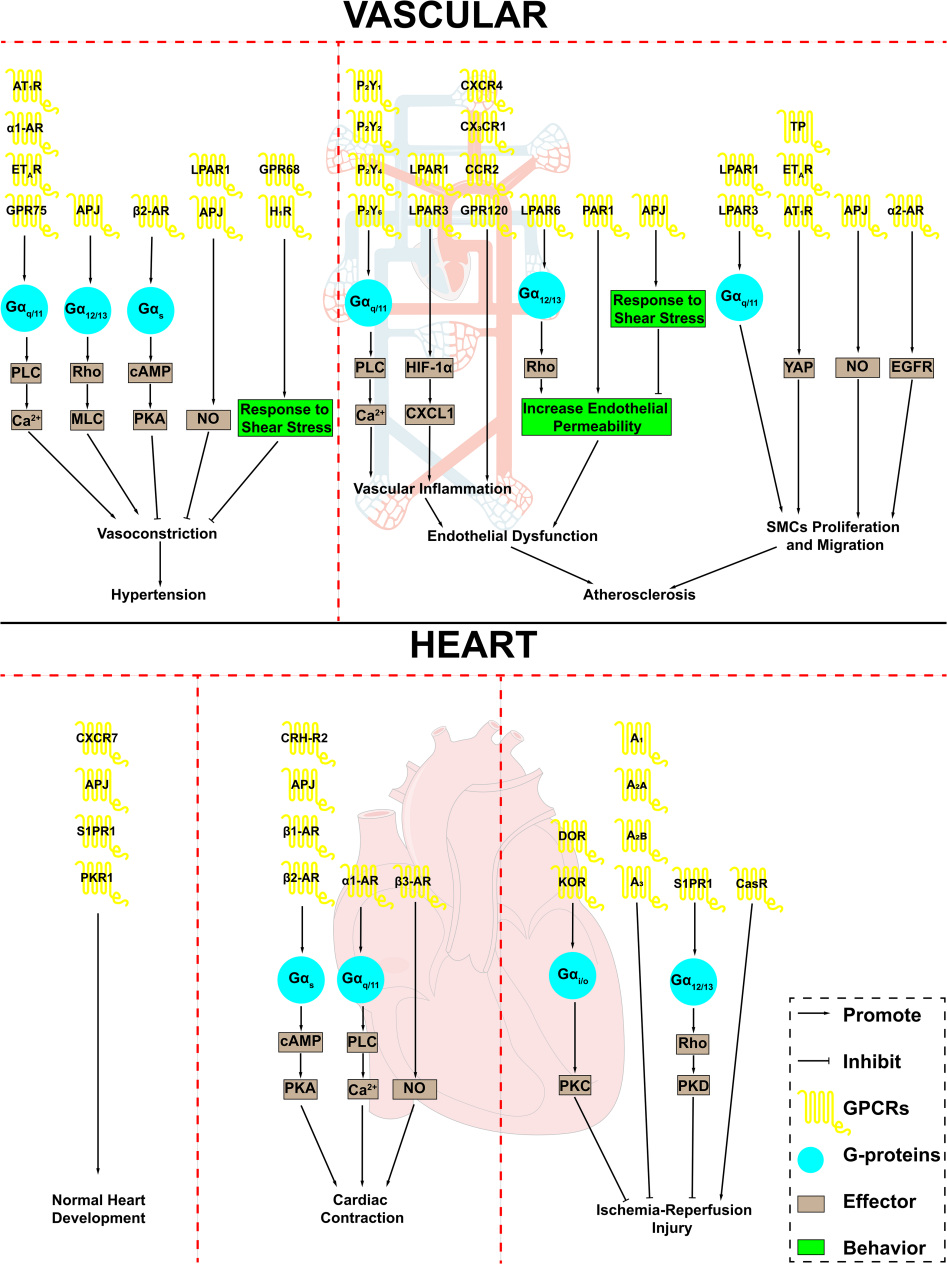
GPCRs in cardiovascular system and cardiovascular disease. GPCRs play an important role in the cardiovascular system and are involved in a variety of cardiovascular disease processes. AT_1_R, angiotensin II type 1 receptor; α1-AR, α1-adrenergic receptor; α2-AR, α2-adrenergic receptor; β1-AR, β1-adrenergic receptor; β2-AR, β2-adrenergic receptor; β3-AR, β3-adrenergic receptor; ET_A_R, endothelin receptor A; APJ, apelin receptor; LPAR1, lysophosphatidic acid receptor 1; LPAR3, lysophosphatidic acid receptor 3; LPAR6, lysophosphatidic acid receptor 6; H_1_R, H_1_ histamine receptor; P_2_Y_1_, purinergic receptor P2Y1; P_2_Y_2_, purinergic receptor P2Y2; P_2_Y_4_, purinergic receptor P2Y4; P_2_Y_6_, purinergic receptor P2Y6; CXCR4, CXC-motif chemokine receptor-4; CCR2, combining CC chemokine receptor 2; PAR1, Protease-activated receptor 1; TP, thromboxane A2 receptor; S1PR1, Sphingosine 1-phosphate receptor 1; PKR1, Prokineticin receptor-1; CRH-R2, corticotropin-releasing hormone receptor 2; DOR, δ opioid receptor; KOR, κ opioid receptor; A1, adenosine A1 receptor; A_2A_, adenosine A2a receptor; A_2B_, adenosine A2b receptor; A3, adenosine A3 receptor; CasR, calcium-sensing receptor; PLC, phospholipase C; MLC, myosin light chain; cAMP, cyclic-3′,5′-adenosine monophosphate; PKA, protein kinase A; NO, nitric oxide; HIF-1α, hypoxia inducible factor 1 subunit alpha; CXCL1, C-X-C motif chemokine ligand 1; YAP, Yes-associated protein; EGFR, epidermal growth factor receptor; SMCs, smooth muscle cells.

Since CVDs are the leading cause of death globally, it is essential to find therapeutic targets for CVDs and develop drugs to treat CVDs. Because of their signal transduction function, GPCRs play a critical role in the occurrence and development of CVDs (Figure 1). In terms of vascular function and disease, GPCRs can receive a variety of extracellular stimuli, including their ligands or mechanical stress, regulate vascular tension and endothelial function, then positively or negatively adjust vascular diseases such as hypertension and atherosclerosis. There are significant gender differences in the occurrence of CVDs, with a higher incidence of CVDs in men compared to women (159). Multiple GPCRs play an integral role in this phenomenon, the most prominent is the G protein-coupled estrogen receptor (GPER), whose activation by estrogen is a key factor in female-specific cardiovascular protection (160). In addition, the orphan receptor GPR37L1 is also involved in the sex differences in CVDs. Mice lacking GPR37L1 exhibited female-specific increases in systolic, diastolic and mean arterial pressure. However, the gender issues on GPCR functions in CVDs are still not clear and further studies are still needed (161–163). In addition, GPCRs regulate the development and function of the heart, and further participate in heart diseases as a target for treatment. Finally, an understanding of the roles of these GPCRs in the cardiovascular system and CVDs will provide new insights into GPCRs and new ideas for fully exploiting the enormous treasure trove of GPCRs.
